# Phyloproteomic Analysis of 11780 Six-Residue-Long Motifs Occurrences

**DOI:** 10.1155/2015/208346

**Published:** 2015-05-31

**Authors:** O. V. Galzitskaya, M. Yu. Lobanov

**Affiliations:** Institute of Protein Research, Russian Academy of Sciences, 4 Institutskaya Street, Pushchino, Moscow Region 142290, Russia

## Abstract

How is it possible to find good traits for phylogenetic reconstructions? Here, we present a new phyloproteomic criterion that is an occurrence of simple motifs which can be imprints of evolution history. We studied the occurrences of 11780 six-residue-long motifs consisting of two randomly located amino acids in 97 eukaryotic and 25 bacterial proteomes. For all eukaryotic proteomes, with the exception of the Amoebozoa, Stramenopiles, and Diplomonadida kingdoms, the number of proteins containing the motifs from the first group (one of the two amino acids occurs once at the terminal position) made about 20%; in the case of motifs from the second (one of two amino acids occurs one time within the pattern) and third (the two amino acids occur randomly) groups, 30% and 50%, respectively. For bacterial proteomes, this relationship was 10%, 27%, and 63%, respectively. The matrices of correlation coefficients between numbers of proteins where a motif from the set of 11780 motifs appears at least once in 9 kingdoms and 5 phyla of bacteria were calculated. Among the correlation coefficients for eukaryotic proteomes, the correlation between the animal and fungi kingdoms (0.62) is higher than between fungi and plants (0.54). Our study provides support that animals and fungi are sibling kingdoms. Comparison of the frequencies of six-residue-long motifs in different proteomes allows obtaining phylogenetic relationships based on similarities between these frequencies: the Diplomonadida kingdoms are more close to Bacteria than to Eukaryota; Stramenopiles and Amoebozoa are more close to each other than to other kingdoms of Eukaryota.

## 1. Introduction

By the middle of the XXth century, it had become clear that all living organisms of cellular texture are divided into two groups or kingdoms, prokaryotes and eukaryotes, according to structural peculiarities of their cells. It was long believed that the terms “prokaryotes” and “bacteria” are synonyms for the same independent evolutionary branch of living organisms. However, about 30 years ago, molecular comparisons of base sequences of ribosomal RNAs provided grounds to divide prokaryotes into at least two independent branches, Eubacteria and Archaebacteria, which differ in their origin [1]. Later, these data were generalized and the term DOMAIN was suggested, which is the branch that has the highest rank in the hierarchic taxonomy [2]. These DOMAINS are Bacteria, Archaea, and Eukaryota.

Protein phylogeny was developed simultaneously with RNA phylogeny [3, 4]. Protein phylogeny is similar to the developed RNA phylogeny because it is based on the division of living organisms into three DOMAINS. RNA and protein phylogenies are based on the alignments of sequences from different organisms, and most phylogenetic methods are based on comparison of protein or nucleic acid sequences in their aligned parts. The conventional tree-building methods for phylogenetic reconstructions are neighbor joining (NJ) [5], maximum parsimony (MP) [6], and maximum likelihood (ML) [7]. Moreover, there is an additional approach as alignment-free phylogeny methods based on k-mer appearance in genomic DNA [8–12].

The understanding of how different major groups of organisms are related to each other and the tracing of their evolution from the common ancestor remains controversial and unsolved. In recent years, the wealth of new information based on a large number of gene and protein sequences has become available. At present, a phylogenetic analysis can be carried out based on either nucleic acid or protein sequences. Nonetheless, the phylogenetic relationship among the kingdoms Animalia, Plantae, and Fungi remains uncertain despite extensive attempts to clarify it. The first hypothesis states that Animalia is more closely related to Plantae [13–15]. The second one supports Plantae and Fungi grouping [16]; the third one, Animalia and Fungi [17–23]. To elucidate evolutionary relationships among different proteomes we will consider the occurrence of some simple motifs which can be imprints of evolution history.

What candidates can be stated as simple motifs? We have done several investigations in this direction. First, by combining the motif discovery and disorder protein segment identification in the Protein Data Bank (PDB: http://www.rcsb.org/), we have compiled the largest database of disordered patterns (171) from the clustered PDB where identity between chains inside a cluster is larger than or equal to 75% using simple rules of selection [21–24]. Second, among these patterns, the patterns with low complexity are more abundant and the length of these motifs is six residues. Third, the patterns with frequent occurrence in proteomes have low complexity (PPPPP, GGGGG, EEEED, HHHH, KKKKK, SSTSS, and QQQQQP), and the type of patterns varies across different proteomes [21]. It is supposed that if an amino acid motif possesses no definite spatial structure in most protein structures, it is likely to be disordered in a protein with an unknown spatial structure [21]. Therefore, the patterns with the length of six residues and low complexity, which are, for example, homorepeats of 20 amino acids, are the major candidates for this role. The length of six residues is important: (1) the experiments performed demonstrated that a minimum repeat size of 6 histidine residues was required for efficient protein translocation to nuclear speckles [25]; (2) six-residue patches affect the folding/aggregation features of proteins, and they are important “words” for the understanding of protein dynamics [26]; (3) nucleation sites are constrained by patches of approximately six residues [27, 28].

It has been found that homorepeats of some amino acids (runs of a single amino acid) occur more frequently than others and the type of homorepeats varies across different proteomes [21]. For example, EEEEEE appears to be the most frequent for all considered proteomes for Chordata, QQQQQQ for Arthropoda, and SSSSSS for Nematoda. A comparative analysis of the number of proteins containing 6-residue-long homorepeats and the 109 disordered selected patterns in 123 proteomes has demonstrated that the correlation coefficients between numbers of proteins are higher inside the considered kingdom than between them [21]. In these proteins a six-residue-long homorepeat occurs at least once for each of the 20 types of amino acid residues and 109 disordered patterns from the library appearing in 9 kingdoms of Eukaryota and 5 phyla of Bacteria.

Here, we present a new phyloproteomic criterion which is based on the peculiarities of amino acid sequences which is an occurrence of some simple motifs which can be imprints of evolution history. In this work, we focus our attention on studying the frequency of six simple amino acid motifs consisting of two randomly located amino acids (11780 motifs) in 122 eukaryotic and bacterial proteomes.

## 2. Materials and Methods

### 2.1. Construction of the Library of Six-Residue-Long Motifs 

We constructed the library of all possible motifs composed of two amino acids, with the assumption that each amino acid could be at any position and at any ratio and that such a motif was six amino acids long [29]. There were 11780 = (2^6^ − 2) · *C*_20_^2^ such motifs in total (excluding two homorepeats for every amino acid pair). The obtained motifs could be divided into three groups. The first group contains the motifs where one of the two amino acids occurs only once and occupies the first or sixth (i.e., outside) position. The second group includes motifs where the second amino acid also occurs once but is inside the motif. The third group contains all the other motifs where each of the two amino acids occurs at least twice and in any order.

### 2.2. Database of Proteomes

We considered 3279 proteomes from the EBI site (ftp://ftp.ebi.ac.uk/pub/databases/SPproteomes/last_release/uniprot/proteomes/). A preliminary analysis showed that the number of proteins with at least one occurrence of homorepeats, 6 residues long, is less than 500 for proteomes with an overall number of residues below 2,500,000. Even so, only 22 proteomes out of 3156 have more than 100 proteins with at least one occurrence of 6-residue homorepeats. These data provided grounds for our research involving only proteomes with an overall number of residues exceeding 2,500,000.

We obtained 122 proteomes taking into account the length of proteomes representing 9 kingdoms of eukaryotes and 5 phyla of Bacteria (see Table  1 in [21]). Unfortunately, only three kingdoms of eukaryotes (Metazoa, Viridiplantae, and Fungi) are given at http://www.ncbi.nlm.nih.gov/Taxonomy. In other cases, the rank of kingdom is missing. In such situations, we chose the highest taxonomic category following from the subkingdom of eukaryotes instead of the kingdom. We chose 97 out of 120 eukaryotic proteomes and a small number of bacterial proteomes. The smallest eukaryotic proteome belongs to* Hemiselmis andersenii*, class Cryptophyta. It is evident that 498 proteins with an overall number of 167,452 amino acid residues are not sufficient for reliable statistics. Historically, the superkingdom of Bacteria is divided into phyla but not kingdoms. We preferred to consider such phyla separately.

Among 97 eukaryotic proteomes, 17 belong to the kingdom of Metazoa or animals:* Homo sapiens* (51778 protein sequences),* Bos taurus* (18405),* Mus musculus*  (42120),* Rattus norvegicus* (28166),* Gallus gallus* (12954),* Danio rerio* (21576), and* Tetraodon nigroviridis* (27836) belong to Chordata phylum;* Drosophila melanogaster* (15101),* Drosophila pseudoobscura* (16000),* Aedes aegypti* (16042),* Anopheles darlingi* (11437), and* Anopheles gambiae* (12455) to arthropods;* Caenorhabditis briggsae* (18531),* Caenorhabditis elegans* (23817), Loa loa (16271), and* Trichinella spiralis* (16040) to nematodes;* Nematostella vectensis* (24435) belongs to Cnidaria phylum.

### 2.3. Calculation of Correlation Coefficient

The vectors of 11780 values for each type of motif are compared between different proteomes. The correlation coefficient (*r*) was calculated using the equation

(1) $$\begin{array}{c} r=\frac{\left(1/n\right)\sum_{i=1}^{n}\left(x_{i}-\overset{-}{x}\right)\left(y_{i}-\overset{-}{y}\right)}{S_{x}S_{y}}, \end{array}$$

where *S*_*x*_ and *S*_*y*_ are the standard deviations for variables *x* and *y*.

For 20 homorepeats, the standard error in determining the correlation coefficient is less than $1/\sqrt{20-2}≅0.24$. The standard error of correlation coefficient is ${se}_{r}=\sqrt{\left(1-r^{2}\right)/\left(n-2\right)}$ where *n* is the number of points; for 109 disordered patterns it is less than $1/\sqrt{109-2}≅0.1$, and for 11780 patterns it is less than 0.01. Therefore, in Tables 3–7 the correlation coefficients range as follows: less than 0.5, from 0.5 to 0.75, and larger than 0.75.

## 3. Results and Discussion

### 3.1. Occurrences of Motifs in 122 Proteomes

We constructed the library of all possible motifs consisting of the two amino acids, with the assumption that each amino acid could be at any position and at any ratio and that such a motif was six amino acid residues long. There were 11780 such motifs in total. The obtained motifs were divided into three groups (see Section 2). The numbers of motifs in the first, second, and third groups were 760 (6%), 1520 (13%), and 9500 (81%), respectively. We estimated the occurrences of these motifs in 122 proteomes.

The most often occurrences of simple motifs for 122 proteomes from the three groups are presented in Table 1. Among the motifs from the first group, the leaders from the human proteome were EEEEED (422 times), DEEEEE (370), LPPPPP (327), APPPPP (264), PLLLLL (251), and PPPPPL (216). It should be noted that such motifs as LPPPPP, PLLLLL, and PPPPPL are not leaders among the occurrences of 122 proteomes (see Table 1). Among the motifs in which one amino acid occurred once and only inside the motif, the leaders from the human proteome were EEEEDE (288), EDEEEE (279), EEDEEE (248), EEEDEE (250), PLPPPP (239), and PPPPLP (207). Among the leaders in which the two amino acids occurred were SGSGSG (135), EEEEDD (157), GPPGPP (162), and RSRSRS (153). The following rare motifs that appeared only in two proteins should be noted for the human proteome: FFFFFN, FFFFFP, CHHHHH, MVVVVV, IHHHHH, WKKKKK, NNNNNS, and IIIIIF from the first group; IIMIII, RRFRRR, YLYYYY, NNCNNN, HHTHHH, and DDQDDD from the second group; and CCCRRR, MMMGGG, TTTDDD, FFSFFS, FFPFFP, VVRVVR, QQKQQK, and DDHDDH from the third group. At the same time, the NNNNNS motif is among the leader motifs for 122 proteomes and it occurs 146 times in the* Drosophila melanogaster* proteome and 473 times in the* Plasmodium falciparum* proteome (Alveolata kingdom). An analogous situation is observed for SNNNNN. It does not occur in the human proteome and appears in 489 proteins for the* Plasmodium falciparum* proteome. PQQQQQ occurs 52 times in the human proteome and 413 times in the* Dictyostelium discoideum* proteome.

In frequently occurring motifs from the* Drosophila melanogaster* proteome, the leading amino acids were glutamine, alanine, and glycine. Among the motifs from the first group, the leaders were QQQQQH (470), HQQQQQ (410), LQQQQQ (359), QQQQQL (359), QQQQQP (276), PQQQQQ (260), QQQQQA (221), AQQQQQ (219), and SAAAAA (224). Among the motifs of the second group, the leaders were QQQQHQ (319), QQQHQQ (297), QQHQQQ (290), QHQQQQ (284), QQQQLQ (243), QLQQQQ (229), and QQLQQQ (218). Among the motifs of the third group, the leaders were GSGSGS (174), SGSGSG (157), HHQQQQ (163), QQQQHH (166), SSGGGG (110), and GGSGSG (105).

Out of 11780 motifs, 865 were not found in 122 proteomes.

**Figure 1:**
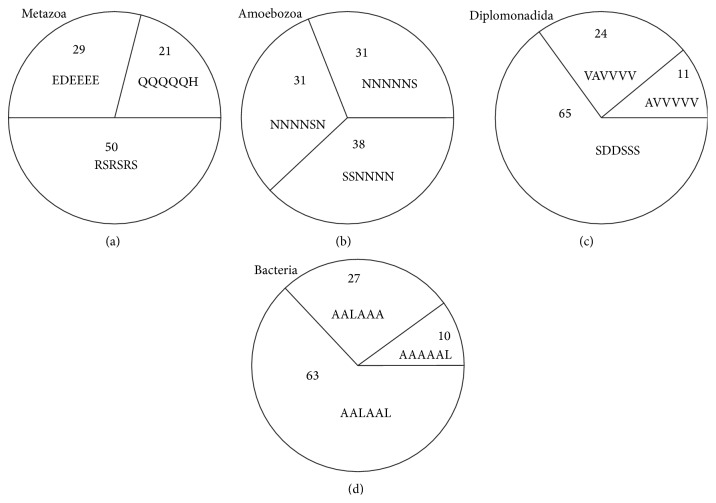
Statistics of occurrence of motifs, six residues long. Statistics of occurrence of motifs, six residues long, consisting of two amino acids in the three groups for 3 kingdoms of Eukaryota and for 5 phyla of Bacteria in percentage terms: (a) the Metazoa kingdom (17 proteomes), (b) the Amoebozoa kingdom (2 proteomes), (c) the Diplomonadida kingdom (3 proteomes), and (d) 26 bacterial proteomes. For each kingdom, the motif with the frequent occurrence in the group is presented.

We estimated the occurrence of the motifs from the three groups in 9 kingdoms of Eukaryota and 5 phyla of Bacteria (see Table 2). Interestingly, for all eukaryotic proteomes with the exception of the Amoebozoa and Diplomonadida kingdoms, the number of proteins containing at least one motif from the first group was about 20%; in the case of motifs from the second and third groups, 30% and 50%, respectively (see Table 2). For bacterial proteomes this relationship is 10%, 27%, and 63%, respectively. One can see that proteomes from the Diplomonadida kingdom are more close to bacterial proteomes than to eukaryotic ones (see Figure 1). It should be noted that diplomonads are a group of flagellates, most of which are parasitic. At the same time, the proteomes from the Amoebozoa kingdom have different statistics: 31%, 31%, and 38%, respectively. For the Metazoa, Amoebozoa, Diplomonadida, and Bacteria kingdoms, the motifs with the frequent occurrence in the groups are presented in Figure 1.

Among animal proteomes, one can see some deviation from the average values for* Nematostella vectensis* (class Anthozoa, phylum Cnidaria): 14%, 27%, and 59%, correspondingly. This is more close to the statistics for the bacterial proteomes. Another deviation from the average values is observed for phylum Arthropoda, especially for class Insecta (29%, 30%, and 41% for* Anopheles darlingi* and 26%, 29%, and 45% for* Anopheles gambiae*).

It should be also noted that the proteins bearing motifs from the third group occurred more frequently than the proteins with motifs from the two other groups only because the third group contained a significantly larger number of motifs (12.5 times as many as in the first group). It might be noted that motifs from the first groups are the simplest, being homorepeats with an adjacent amino acid. Motifs from the second group are homorepeats with an inclusion of the other amino acid. Meanwhile, members of the third group can hardly be derived from homorepeats. The most frequent motifs are the ones most closely resembling homorepeats, that is, the motifs from the first group, whereas the motifs from the second group occur somewhat more rarely, and the motifs not resembling homorepeats are the rarest of all. Each proteome contains its characteristic leading motifs, and it is apparent that the amino acids foremost among six amino acid repeats occur most often.

### 3.2. Construction of Matrices of Correlation Coefficients for Proteins Containing Simple Motifs in the Studied Proteomes

For each proteome, we calculated a set of 11780 values reflecting the number of proteins containing at least one simple motif, 6 residues long. Then considering all possible pairs of proteomes, the correlation coefficients between the 11780 values have been calculated which allowed us to construct a matrix of correlation coefficients (see Table 3). As a rule, the correlation coefficients are higher inside the studied kingdom than between them. A similar conclusion follows from considering the occurrence of motifs from the three groups (see Tables 4, 5, and 6). “∗∗” in Tables 3–7 is used to show the correlation higher than 75%, and “∗” is used to show the correlation from 50% to 75%. Usually, the correlation coefficients are higher inside the considered kingdom than between them. The highest correlation is observed for the Amoebozoa kingdom in all cases (see Tables 3–6).

Most of the theories suggest that colonial naked choanoflagellate-like protists gave rise to first animals, while chitinous thecate choanoflagellate-like protists gave rise to first fungi [30, 31]. In the case of occurrence of the motifs from the first and second groups, we obtained a high correlation between the Choanoflagellida and Fungi kingdoms (0.67 and 0.61) compared to between the Choanoflagellida and animals kingdoms (0.61 and 0.54) (see Tables 4–6).

We averaged the correlation coefficients over all proteomes from the studied kingdoms. The averaged correlation coefficient is low inside such a kingdom as Metazoa (see Table 3). We decided to analyze in more detail the proteomes from the Metazoa kingdom. If the correlation coefficients for animal proteomes only (see Table 7) are to be considered, four clusters can be selected with high correlation between the numbers of proteins where a simple motif, 6 residues long, appears at least once. The first cluster corresponds to the phylum Chordata (7 proteomes), the second to Arthropoda (5 proteomes), the third to Nematoda (4 proteomes), and the fourth to Cnidaria (only 1 proteome). Again one can see that the correlation coefficients are higher inside the considered phylum than between them.

In Table 7 one can see that the correlation coefficient between zebrafish,* Danio rerio*, and pufferfish,* Tetraodon nigroviridis*, is 0.72, while on the other hand that between* D. rerio* and starlet sea anemone,* Nematostella vectensis*, is 0.77 and those between* D. rerio* and two nematodes,* Caenorhabditis elegans* and* C. briggsae*, are 0.73 and 0.80, respectively. The correlation coefficients between* T. nigroviridis* and other vertebrates are 0.70–0.75, while those between* D. rerio* and other vertebrates, except for* T. nigroviridis*, are 0.80–0.86. These values suggest that the pattern of six-residue-long motifs in* T. nigroviridis* has changed very rapidly after the separation of the lineages of pufferfish (belongs to a family of primarily marine and estuarine fish) and zebrafish (a tropical freshwater fish). This fact is not surprising in light of the last data, that horses were evolutionarily closest to Brandt's bats (*Myotis brandtii*); their divergence occurred about 81.7 million years ago, which is close to the time of the adaptive radiation of the class Mammalia [32].

In the case of the occurrence of simple motifs (all 11780 and 9500 for the third group), there is no high correlation (larger than 0.5) between eukaryotic and bacterial proteomes. Among the correlation coefficients for eukaryotic proteomes, there is a high correlation between the animal and Fungi kingdoms (0.62) compared to between the fungi and plants (0.54). This is valid also in the case of consideration of the correlation coefficients for the occurrence of the motifs from the three groups separately (see Tables 4–6). Moreover, this result agrees with the results obtained by us after analysis of loops in elongation factors EF1A using the novel informative characteristic called the “loops” method [20]. The method is based on the ability of amino acid sequences to form flexible loops in protein structure. Each kingdom displayed variations in the number of loops and their location within the three EF1A domains. It has been found that animals and fungi are sibling kingdoms [20].

## 4. Conclusions

One can see that some simple motifs have been maintained throughout evolution and that in the studied 122 eukaryotic and bacterial proteomes the most frequent motifs are specific for each proteome. The ratio between occurrences of the simple motifs from the three groups is practically the same for the eukaryotic proteomes. The other relationship between occurrences of the motifs is observed for the bacterial proteomes. The question about specificity of these motifs is more important for biological functioning. Our study provides support that animals and fungi are sibling kingdoms.

## Figures and Tables

**Table 1:** 11780 motifs that frequently occur in 123 proteomes.

11780		The first group		The second group		The third group	
EEEEED	6744	EEEEED	6744	EDEEEE	4248	APAPAP	3543
QQQQQP	6300	QQQQQP	6300	STSSSS	4166	DDEEEE	3464
DEEEEE	6165	DEEEEE	6165	NNNNSN	4030	SGSGSG	3423
TSSSSS	6135	TSSSSS	6135	EEEEDE	3995	PAPAPA	3392
SGGGGG	6117	SGGGGG	6117	NSNNNN	3992	EEEEDD	3292
AAAAAG	5863	AAAAAG	5863	EEDEEE	3959	GSGSGS	3240
PSSSSS	5813	PSSSSS	5813	SSSSTS	3953	DEDEDE	3127
NNNNNS	5811	NNNNNS	5811	GGGGSG	3934	EDEDED	3045
QQQQQH	5798	QQQQQH	5798	AAVAAA	3768	RSRSRS	2983
SSSSST	5780	SSSSST	5780	AAAVAA	3758	DDDDEE	2953
DDDDDE	5611	DDDDDE	5611	GSGGGG	3690	EEEDDD	2845
SNNNNN	5585	SNNNNN	5585	SSTSSS	3660	DDDEEE	2822
ASSSSS	5581	ASSSSS	5581	SSSTSS	3652	RGRGRG	2817
SAAAAA	5405	SAAAAA	5405	EEEDEE	3627	EEDDDD	2754
APPPPP	5325	APPPPP	5325	AAAAVA	3616	AAAAGG	2743
AAAAAS	5322	AAAAAS	5322	GGGSGG	3556	EDEDEE	2651
AAAAAV	5277	AAAAAV	5277	SPSSSS	3459	DDEDED	2570
GGGGGS	5118	GGGGGS	5118	NNSNNN	3429	RGGRGG	2537
GGGGGA	4862	GGGGGA	4862	NNNSNN	3418	DEDEDD	2489
PQQQQQ	4819	PQQQQQ	4819	AVAAAA	3391	SSSSTT	2448

**Table 2:** Occurrence of 11780 motifs from the three groups in 9 kingdoms of Eukaryota and for 5 phyla of Bacteria in percentage terms.

Kingdom	<x>	Error	<x>	Error	<x>	Error
	First group		Second group		Third group	
Metazoa (17)	21	3	29	1	50	4
Viridiplantae (5)	21	4	28	2	51	5
Stramenopiles (1)	28	—	32	—	41	—
Choanoflagellida (1)	18	—	27	—	55	—
Euglenozoa (4)	22	3	29	2	49	4
Alveolata (6)	23	4	29	1	48	5
Amoebozoa (2)	31	1	31	0	38	2
Diplomonadida (3)	11	1	24	1	65	2
Fungi (58)	18	3	28	1	53	4

Bacteria (25)	10	1	27	2	63	3

All 11780 motifs	6	0	13	0	81	0

**Table 3:** Averaged correlation coefficients (in percentage terms) between numbers of proteins where a simple motif, six residues long, from the whole set of **11780** motifs appears at least once in 9 kingdoms of Eukaryota and 5 phyla of Bacteria.

Metazoa (17)	Viridiplantae (5)	Stramenopiles (1)	Choanoflagellida (1)	Euglenozoa (4)	Alveolata (6)	Amoebozoa (2)	Diplomonadida (3)	Fungi (58)	Acidobacteria (1)	Actinobacteria (14)	Proteobacteria (8)	Bacteroidetes (2)	Chloroflexi (1)	
65^*^	49	46	29	47	22	30	35	62^*^	27	25	27	26	24	Metazoa (17)
49	61^*^	60^*^	28	47	14	13	27	54^*^	41	43	42	21	22	Viridiplantae (5)
46	60^*^	—	20	52^*^	8	8	16	41	49	48	44	23	17	Stramenopiles (1)
29	28	20	—	30	7	13	23	32	20	21	22	12	15	Choanoflagellida (1)
47	47	52^*^	30	47	10	17	27	45	35	39	35	24	22	Euglenozoa (4)
22	14	8	7	10	69^*^	37	8	25	−1	−1	0	13	3	Alveolata (6)
30	13	8	13	17	37	90^**^	13	35	−1	−1	−1	5	3	Amoebozoa (2)
35	27	16	23	27	8	13	68^*^	38	21	22	22	25	27	Diplomonadida (3)
62^*^	54^*^	41	32	45	25	35	38	71^*^	28	26	28	21	21	Fungi (58)

27	41	49	20	35	−1	−1	21	28	—	70^*^	67^*^	32	39	Acidobacteria (1)
25	43	48	21	39	−1	−1	22	26	70^*^	87^**^	74^*^	29	38	Actinobacteria (14)
27	42	44	22	35	0	−1	22	28	67^*^	74^*^	72^*^	29	39	Proteobacteria (8)
26	21	23	12	24	13	5	25	21	32	29	29	39	39	Bacteroidetes (2)
24	22	17	15	22	3	3	27	21	39	38	39	39	—	Chloroflexi (1)

**Table 4:** Averaged correlation coefficients (in percentage terms) between numbers of proteins where a simple motif, six residues long, from the first group (760 motifs) appears at least once in 9 kingdoms of Eukaryota and 5 phyla of Bacteria.

Metazoa (17)	Viridiplantae (5)	Stramenopiles (1)	Choanoflagellida (1)	Euglenozoa (4)	Alveolata (6)	Amoebozoa (2)	Diplomonadida (3)	Fungi (58)	Acidobacteria (1)	Actinobacteria (14)	Proteobacteria (8)	Bacteroidetes (2)	Chloroflexi (1)	
64^*^	49	45	61^*^	50^*^	13	26	37	65^*^	34	29	35	29	32	Metazoa (17)
49	62^*^	61^*^	60^*^	48	6	7	28	54^*^	48	47	52^*^	22	22	Viridiplantae (5)
45	61^*^	—	45	53^*^	0	1	14	40	68^*^	64^*^	63^*^	28	20	Stramenopiles (1)
61^*^	60^*^	45	—	60^*^	7	28	35	67^*^	42	46	47	26	29	Choanoflagellida (1)
50^*^	48	53^*^	60^*^	51^*^	3	16	30	49	47	49	48	31	32	Euglenozoa (4)
13	6	0	7	3	74^*^	29	4	16	−6	−6	−5	8	−1	Alveolata (6)
26	7	1	28	16	29	92^**^	12	33	−5	−5	−5	1	−1	Amoebozoa (2)
37	28	14	35	30	4	12	67^*^	40	17	16	19	29	31	Diplomonadida (3)
65^*^	54^*^	40	67^*^	49	16	33	40	74^*^	29	25	32	20	22	Fungi (58)

34	48	68^*^	42	47	−6	−5	17	29	—	75^*^	73^*^	40	38	Acidobacteria (1)
29	47	64^*^	46	49	−6	−5	16	25	75^*^	90^**^	76^**^	30	28	Actinobacteria (14)
35	52^*^	63^*^	47	48	−5	−5	19	32	73^*^	76^**^	74^*^	33	33	Proteobacteria (8)
29	22	28	26	31	8	1	29	20	40	30	33	52^*^	57^*^	Bacteroidetes (2)
32	22	20	29	32	−1	−1	31	22	38	28	33	57^*^	—	Chloroflexi (1)

**Table 5:** Averaged correlation coefficients (in percentage terms) between numbers of proteins where at least once a simple motif, six residues long, from the second group (1520 motifs) appears in 9 kingdoms of Eukaryota and 5 phyla of Bacteria.

Metazoa (17)	Viridiplantae (5)	Stramenopiles (1)	Choanoflagellida (1)	Euglenozoa (4)	Alveolata (6)	Amoebozoa (2)	Diplomonadida (3)	Fungi (58)	Acidobacteria (1)	Actinobacteria (14)	Proteobacteria (8)	Bacteroidetes (2)	Chloroflexi (1)	
64^*^	45	42	54^*^	47	20	23	39	63^*^	26	24	27	26	27	Metazoa (17)
45	64^*^	62^*^	58^*^	50^*^	10	5	28	52^*^	51^*^	52^*^	52^*^	22	24	Viridiplantae (5)
42	62^*^	—	46	55^*^	6	2	16	40	62^*^	57^*^	56^*^	22	23	Stramenopiles (1)
54^*^	58^*^	46	—	55^*^	11	23	32	61^*^	44	51^*^	52^*^	28	29	Choanoflagellida (1)
47	50^*^	55^*^	55^*^	50	10	13	32	48	41	46	45	27	26	Euglenozoa (4)
20	10	6	11	10	66^*^	40	6	23	−3	−4	−3	13	−1	Alveolata (6)
23	5	2	23	13	40	90^**^	10	30	−4	−4	−4	0	−3	Amoebozoa (2)
39	28	16	32	32	6	10	72^*^	42	17	19	20	25	31	Diplomonadida (3)
63^*^	52^*^	40	61^*^	48	23	30	42	71^*^	28	26	29	19	19	Fungi (58)

26	51^*^	62^*^	44	41	−3	−4	17	28	—	70^*^	69^*^	33	40	Acidobacteria (1)
24	52^*^	57^*^	51^*^	46	−4	−4	19	26	70^*^	92^**^	80^**^	30	35	Actinobacteria (14)
27	52^*^	56^*^	52^*^	45	−3	−4	20	29	69^*^	80^**^	77^**^	32	38	Proteobacteria (8)
26	22	22	28	27	13	0	25	19	33	30	32	47	56^*^	Bacteroidetes (2)
27	24	23	29	26	−1	−3	31	19	40	35	38	56^*^	—	Chloroflexi (1)

**Table 6:** Averaged correlation coefficients (in percentage terms) between numbers of proteins where a simple motif, six residues long, from the third group (9500 motifs) appears at least once in 9 kingdoms of Eukaryota and 5 phyla of Bacteria.

Metazoa (17)	Viridiplantae (5)	Stramenopiles (1)	Choanoflagellida (1)	Euglenozoa (4)	Alveolata (6)	Amoebozoa (2)	Diplomonadida (3)	Fungi (58)	Acidobacteria (1)	Actinobacteria (14)	Proteobacteria (8)	Bacteroidetes (2)	Chloroflexi (1)	
59^*^	44	44	23	37	25	31	31	56^*^	25	26	25	21	22	Metazoa (17)
44	58^*^	54^*^	22	41	17	15	26	53^*^	36	40	38	17	22	Viridiplantae (5)
44	54^*^	—	15	47	11	11	16	41	43	45	39	18	16	Stramenopiles (1)
23	22	15	—	24	6	10	21	25	15	16	16	8	12	Choanoflagellida (1)
37	41	47	24	40	10	14	23	38	30	34	29	17	19	Euglenozoa (4)
25	17	11	6	10	61^*^	38	8	28	0	−1	0	13	5	Alveolata (6)
31	15	11	10	14	38	88^**^	13	36	0	1	0	8	6	Amoebozoa (2)
31	26	16	21	23	8	13	67^*^	33	22	24	24	21	24	Diplomonadida (3)
56^*^	53^*^	41	25	38	28	36	33	68^*^	26	27	27	18	21	Fungi (58)

25	36	43	15	30	0	0	22	26	—	69^*^	65^*^	28	38	Acidobacteria (1)
26	40	45	16	34	−1	1	24	27	69^*^	84^**^	73^*^	28	40	Actinobacteria (14)
25	38	39	16	29	0	0	24	27	65^*^	73^*^	71^*^	26	40	Proteobacteria (8)
21	17	18	8	17	13	8	21	18	28	28	26	30	30	Bacteroidetes (2)
22	22	16	12	19	5	6	24	21	38	40	40	30	—	Chloroflexi (1)

**Table 7:** Averaged correlation coefficients (in percentage terms) between numbers of proteins where a simple motif, six residues long, appears at least once in 17 animal proteomes (kingdom Metazoa).

Phylum	Proteome	*H. sapiens *	*B. taurus *	*M. musculus *	*R. norvegicus *	*G. gallus *	*D. rerio *	*T. nigroviridis *	*D. melanogaster *	*D. pseudoobscura *	*A. aegypti *	*A. darlingi *	*A. gambiae *	*C. briggsae *	*C. elegans *	*L. loa *	*T. spiralis *	*N. vectensis *
Chordata	*H. sapiens *		**95** ^**^	**96** ^**^	**95** ^**^	**89** ^**^	**80** ^**^	**73** ^*^	**53** ^*^	**53** ^*^	**60** ^*^	**48**	**63** ^*^	**70** ^*^	**65** ^*^	**42**	**54** ^*^	**68** ^*^
	*B. taurus *	**95** ^**^		95^**^	95^**^	89^**^	80^**^	70^*^	**49**	49	57^*^	44	60^*^	68^*^	**62** ^*^	38	50^*^	68^*^
	*M. musculus *	**96** ^**^	95^**^		97^**^	90^**^	82^**^	75^**^	**56** ^*^	56^*^	64^*^	52^*^	66^*^	71^*^	**67** ^*^	44	57^*^	70^*^
	*R. norvegicus *	**95** ^**^	95^**^	97^**^		90^**^	83^**^	72^*^	**51** ^*^	50^*^	61^*^	46	61^*^	71^*^	**66** ^*^	43	53^*^	70^*^
	*G. gallus *	**89** ^**^	89^**^	90^**^	90^**^		86^**^	71^*^	**49**	48	61^*^	46	59^*^	74^*^	**68** ^*^	42	55^*^	73^*^
	*D. rerio *	**80** ^**^	80^**^	82^**^	83^**^	86^**^		72^*^	**50** ^*^	48	68^*^	49	60^*^	80^**^	**73** ^*^	48	60^*^	77^**^
	*T. nigroviridis *	**73** ^*^	70^*^	75^**^	72^*^	71^*^	72^*^		**46** ^*^	45	54^*^	43	56^*^	61^*^	**58** ^*^	50^*^	52^*^	59^*^

Arthropoda	*D. melanogaster *	**53** ^*^	**49**	**56** ^*^	**51** ^*^	**49**	**50** ^*^	**46**		**96** ^**^	**87** ^**^	**92** ^**^	**87** ^**^	**57** ^*^	**67** ^*^	**48**	**71** ^*^	**44**
	*D. pseudoobscura *	**53** ^*^	49	56^*^	50^*^	48	48	45	**96** ^**^		84^**^	91^**^	87^**^	53^*^	**63** ^*^	47	71^*^	42
	*A. aegypti *	**60** ^*^	57^*^	64^*^	61^*^	61^*^	68^*^	54^*^	**87** ^**^	84^**^		86^**^	87^**^	73^*^	**79** ^**^	54^*^	76^**^	57^*^
	*A. darlingi *	**48**	44	52^*^	46	46	49	43	**92** ^**^	91^**^	86^**^		91^**^	53^*^	**62** ^*^	51^*^	75^**^	40
	*A. gambiae *	**63** ^*^	60^*^	66^*^	61^*^	59^*^	60^*^	56^*^	**87** ^**^	87^**^	87^**^	91^**^		62^*^	**69** ^*^	48	72^*^	50^*^

Nematoda	*C. briggsae *	**70** ^*^	68^*^	71^*^	71^*^	74^*^	80^**^	61^*^	**57** ^*^	53^*^	73^*^	53^*^	62^*^		**90** ^**^	52^*^	64^*^	71^*^
	*C. elegans *	**65** ^*^	**62** ^*^	**67** ^*^	**66** ^*^	**68** ^*^	**73** ^*^	**58** ^*^	**67** ^*^	**63** ^*^	**79** ^**^	**62** ^**^	**69** ^**^	**90** ^**^		**52** ^*^	**67** ^*^	**66** ^*^
	*L. loa *	**42**	38	44	43	42	48	50^*^	**48**	47	54^*^	51^*^	48	52^*^	**52** ^*^		68^*^	46
	*T. spiralis *	**54** ^*^	50^*^	57^*^	53^*^	55^*^	60^*^	52^*^	**71** ^*^	71^*^	76^**^	75^**^	72^*^	64^*^	**67** ^*^	68^*^		53^*^

Cnidaria	*N. vectensis *	**68** ^*^	68^*^	70^*^	70^*^	73^*^	77^**^	59^*^	**44**	42	57^*^	40	50^*^	71^*^	**66** ^*^	46	53^*^	
